# Evaluating DNA Extraction Methods for Community Profiling of Pig Hindgut Microbial Community

**DOI:** 10.1371/journal.pone.0142720

**Published:** 2015-11-11

**Authors:** Yang Lu, Philip Hugenholtz, Damien John Batstone

**Affiliations:** 1 Advanced Water Management Centre, The University of Queensland, St. Lucia, Brisbane, Queensland, Australia; 2 Australian Centre for Ecogenomics, School of Chemistry and Molecular Biosciences and Institute of Molecular Bioscience, The University of Queensland, St. Lucia, Brisbane, Queensland, Australia; U.S. Geological Survey, UNITED STATES

## Abstract

Recovery of high quality PCR-amplifiable DNA has been the general minimal requirement for DNA extraction methods for bulk molecular analysis. However, modern high through-put community profiling technologies are more sensitive to representativeness and reproducibility of DNA extraction method. Here, we assess the impact of three DNA extraction methods (with different levels of extraction harshness) for assessing hindgut microbiomes from pigs fed with different diets (with different physical properties). DNA extraction from each sample was performed in three technical replicates for each extraction method and sequenced by 16S rRNA amplicon sequencing. Host was the primary driver of molecular sequencing outcomes, particularly on samples analysed by wheat based diets, but higher variability, with one failed extraction occurred on samples from a barley fed pig. Based on these results, an effective method will enable reproducible and quality outcomes on a range of samples, whereas an ineffective method will fail to generate extract, but host (rather than extraction method) remains the primary factor.

## Introduction

The vertebrate hindgut microbiome is critical to host organism nutrition, health, and welfare, including control of infectious disease [1–2]. Faecal samples are used as a simple, non-invasive method of sampling this community [3]. While the hindgut microbiome has been extensively assessed in humans [4], it is also highly important in other animals, including domestic and commercial livestock [5]. This is not only for commercial and welfare reasons, but because large animals such as pigs are being increasingly used as models for human microbiomes [6]. The most common culture-independent method for analysing microbial communities is 16S rRNA amplicon profiling [7] which can be affected by the DNA extraction method [8]. Several studies have attempted to develop or validate DNA extraction methods suitable for faecal samples. Clement et al. [9] modified the UltraClean Soil DNA kit (Mo Bio Laboratories, Solana Beach, CA, USA) with dry lysis tubes and a second DNA wash step to produce a high yield of PCR-quality DNA from human faeces. Tang et al. [10] also described a modified method, utilizing hexadecyltrimethylammonium bromide (CTAB), salt, polyvinylpyrrolidone and beta-mercaptoethanol for cell lysis and chloroform for DNA isolation, producing notably better results than the QIAamp DNA stool mini kit. Salonen et al. [11] also concluded that a DNA extraction method using repeated bead beating [12] can generate up to 35-fold increase on DNA yield than other extraction method with non or less mechanical cell lysis.

DNA yield is most commonly used as a proxy for method quality, the assumption being that a higher yield is more representative of the community under study. However, the severity of extraction is an important factor affecting the representativeness and reproducibility of extraction methods. Overly harsh methods while generally producing higher yields can potentially degrade the DNA of sensitive organisms (e.g. *Bacteroidetes*, [13]), while excessively gentle methods can fail to extract from gram-positive organisms with thick cell walls (e.g. *Clostridium*, [11]). Reproducibility of extraction can also be an issue, particularly in livestock faecal samples, which can have a high degree of heterogeneity due to the faecal matrix, particularly where diet is varied.

The development of next-generation sequencing (NGS) provides a powerful tool to increase depth and resolution of community analysis [7] and NGS has been applied for microbial profiling of faecal sample from humans [14–16] or animals such as swine [5], white rhinoceros [17] and horses [18]. Due to its enhanced depth and resolution, NGS techniques can be more influenced or likely to detect artefacts due to DNA extraction methods, and hence analysis of the impacts of extraction is an important consideration. Recently, the variations resulting from choice of extraction method were shown to be significant in NGS study on human gut [13] but negligible on insects gut [19], while this remains unclear on livestock gut.

Here, we evaluate three commonly used pig faecal DNA extraction methods (FastDNA SPIN kit, PowerSoil kit and a previous reported protocol utilizing CTAB) using NGS sequencing. All three methods gave comparable results despite striking differences in DNA yields.

## Materials and Methods

### Pig Faecal samples

Fresh whole faecal samples were collected from three pigs aged 11 weeks, as part of a larger metabolic trial being conducted at the Advanced Animal Science facility of the University of Queensland (Gatton, Queensland, Australia) with permission from the University of Queensland. Animal ethics approval for the larger metabolic trial (from which faecal samples were obtained) was provided by the Staff Access Animal Ethics Committee, Argri-Science Queensland. Sampling procedures were reviewed and specifically approved as part of the approval.

Faecal samples were collected in sterile bags and stored on ice, immediately taken to the laboratory and subsequently frozen at -20°C. No invasive sampling or sacrificing was involved in sample collection. Pig diets were the three most common commercial Australian carbohydrate based diets and are provided in supplementary material (S1 Table) with mainly wheat fines in pig A (72%) and pig B (65%). The diet for pig C was replaced with barley fines (48%).

### DNA extraction

Samples were homogenized to mix the liquid, mud layers and solid settlement and subsampled. DNA was extracted from 0.3 g aliquots in triplicate using two commercially-available kits: FastDNA SPIN Kit for Soil (MP Biomedicals, Santa Ana, California, US) referred as FAS, PowerSoil^®^ DNA Isolation Kit (MoBio Laboratories, Carlsbad, CA, US) referred as POW and one additional protocol utilizing CTAB adapted from Tang et al. [10] referred as CON. The concentration of each eluted DNA was measured by Nanodrop spectrometer (Thermo Scientific, US) as well as the purity (indicated as 260/280 ratio). The extracted DNA was loaded on a 1% agarose gel to identify extent of DNA degradation.

#### FAS method

DNA extraction with FAS was performed according to manufacturer’s instruction with modification on the bead beating time and additional pre-elution incubation (details below). Sample aliquot was added into the lysing matrix with lysis buffer supplied. Lysing matrix was blended with mini Bead Beater (BioSpec, Bartlesville, US) at 4,800 oscillations per minute for 60 seconds. After being cooled to 4°C, tubes were centrifuged at 13,000 x *g* for 15 minutes to pellet debris. Supernatant was transferred to 250 μL protein precipitation solution and mixed with 1 mL binding matrix. The mixture of binging matrix and DNA was then filtered and washed with 500 μL SEWS-M supplied in the kit. Additional incubation at 50°C for 5 minutes was performed before the final elution. 50 μL RNAnase-free water was used to elute the DNA from filter.

#### POW method

DNA extraction with POW was performed according to the manufacturer’s instructions with modifications of bead beating time and additional pre-elution incubation. Sample aliquot was added into the lysing matrix with lysis buffer supplied. Lysing matrix was then blended with mini Bead Beater (BioSpec, Bartlesville, US) at 4,800 oscillations per minute for 60 seconds. The remaining steps (including protein removal and DNA washing) were performed as recommended. DNA was also eluted with 50 μL RNAnase-free water with pre-elution incubation at 50°C for 5 minutes.

#### CON method

The conventional DNA extraction method used in this study was described by Tang et al. (2008), in which autoclaved beads (0.5 g, 0.3 mm in diameter) were mixed with 570 μL buffer TE in capped tubes. Sample aliquot was then added. After bead beating at 4,800 oscillations per minute for 60 seconds, 5 μL 10% SDS and 3 μL Proteinase K was added to the tube. Tubes were incubated at 37°C for 1 hour and then at 65°C for 10 minutes. Supernatant (600 μL) was transferred to a clean autoclaved 1.5 mL tube with 100 μL 5M NaCl and 80 uL CTAB (65°C). Following this, Phenol:Chlorophom:Isoanyl-alcohol (800 uL, 25:24:2) were added to the tube and centrifuged at 13,000 x *g* for 5 minutes. The aqueous phase was then transferred to a new autoclaved Eppendorf tube. An equal volume of chloroform/Isoamyl-alcohol (50/50 v/v) was then added and mixed. After centrifuging at 13,000 x *g* for 5 minutes, the aqueous phase was then transferred to another autoclaved tube with 300 μL isopropanol. This was incubated at -20°C for 30 minutes. After centrifuging at 4°C, 13,000 x *g* for half an hour, the supernatant was removed and 500 μL 70% ethanol was added. The tube was again centrifuged at 4°C, 13,000 x *g* for 5 minutes. The supernatant was then removed. After the pellet was air dried, DNA pellet was resuspended in 50 μL RNAnase-free water.

### Pyrosequencing and data analysis

DNA samples (300 ng each) were provided to Australian Centre for Ecogenomics (ACE) for pyrosequencing analysis. The primers used for pyrosequencing were 926f (5’-AAACTYAAAKGAATTGACGG-3’) [20] and 1392r (5’-ACGGGCGGTGTGTAC-3’) [21] targeting V6-V8 regions of the SSU rRNA gene with Roche 454 GS FLX sequencer (Roche, Switzerland).

Pyrosequencing results were analysed through the ACE Pyrosequencing Pipeline (https://github.com/minillinim/APP). Sequences reads were split according to barcodes in QIIME v1.8.0 [22]. De-multiplexed sequences were then trimmed to 250bp and de-noised by ACACIA [23]. Sequences with ≥97% similarity were assigned to operational taxonomic units (OTUs) by CD-HIT-OTU [24–25] and aligned by Pynast [26]. Each sequence was then classified using BlastTaxonAssigner in QIIME against the Greengenes database (2012 October release). Weighted UniFrac distances [27] were also calculated in QIIME.

Non-normalized OTU tables and rarefaction curves were generated by QIIME. An in-house script Normaliser (https://github.com/minillinim/Normaliser) was used to find a centroid normalized OTU table. The normalized OTU table was imported into R (version 3.0.1, [28]) to generate multidimensional scaling analysis using Bray-Curtis dissimilarity method with function metaMDS in Package “vegan” [29]. Analysis of variance (ANOVA) was performed on the OTU table with function aov in package “stats” [28]. Power analysis of ANOVA was performed with function pwr.anova.test in package “pwr” in R [30] with number of groups as 3, and observations per group of 9. Eta-squared effect size was calculated according to [31] as: sum of squares between groups / total sum of squares. Analysis of similarity (ANOSIM) was performed in QIIME [22]. De-multiplexed pyrosequencing data were deposited in GenBank under BioProject PRJNA286807.

## Results and Discussion

### DNA quantity and purity

**Fig 1:**
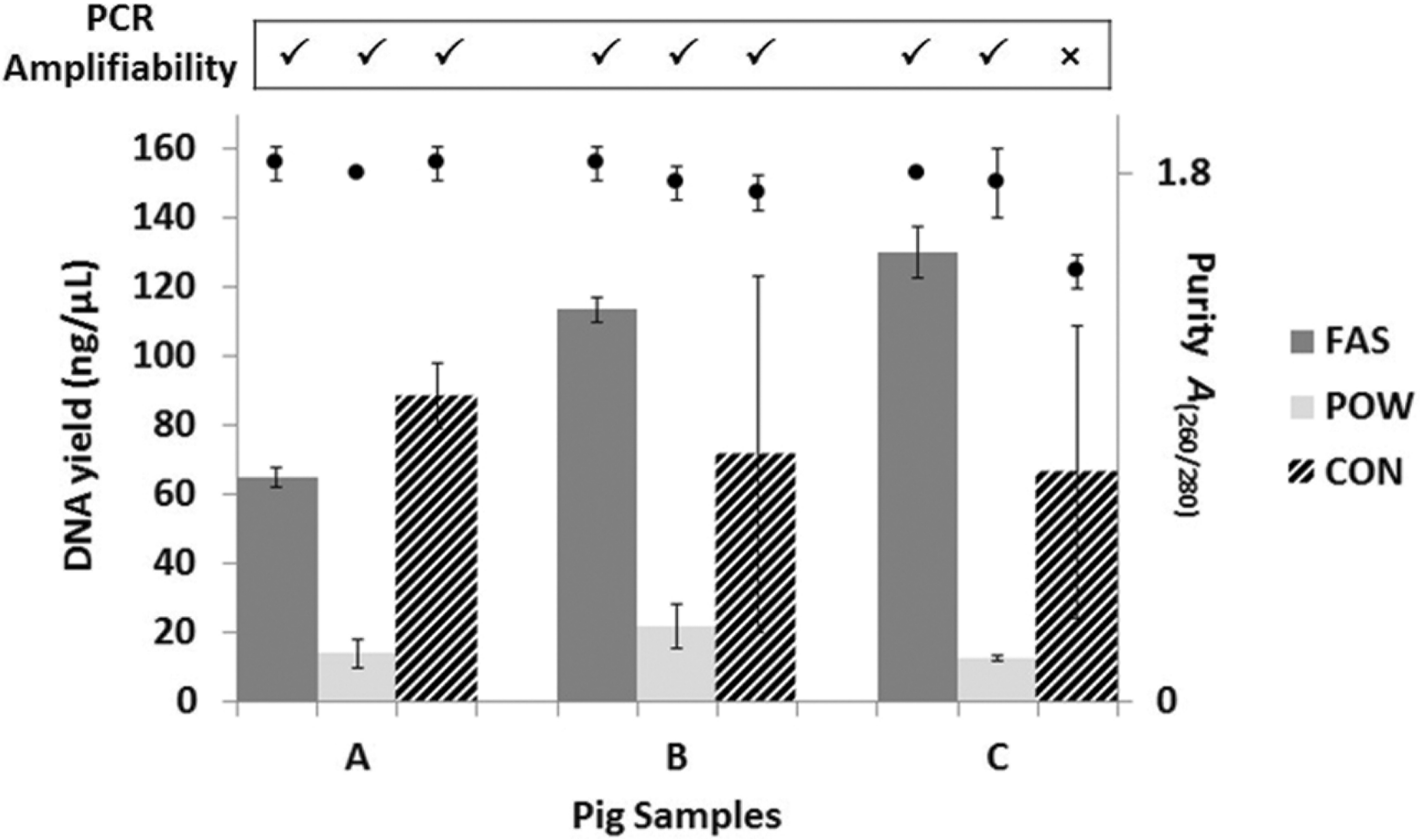
Yield (bars), purity (●) and PCR amplifiability of extracted DNA from pigs A, B, and C by different extraction methods FAS, POW and CON. FAS method yields more pure and amplifiable DNA than other methods. Yield (bars) is plotted on the left y-axis, purity (**●**) is plotted on the right y-axis. Error bars represent standard deviation. PCR amplifiabilities are indicated as “✓” for successful amplification or “×” for failed amplification).

DNA of faecal samples collected from pig A and B fed with mainly wheat fines and pig C fed with mainly barley, were extracted using two commercial kits (FAS and POW) and one conventional method (CON). With the exception of pig C extracted by CON, all other samples recovered high purity DNA template with A_260_/A_280_ ratios being close to 1.8 (Fig 1). The amount of DNA extracted from FAS was the highest with up to two and ten times the concentration of DNA compared to CON and POW respectively. There was high variability in concentration of DNA extracted from CON in both pig B and C replicates as shown by a large error bar in Fig 1. POW gave consistently lowest but reproducible DNA concentration (10–20 ng·μL^-1^) in all samples. FAS resulted in discrete high molecular weight bands from pig A and B, but with degraded DNA from pig C (S1 Fig). Clear single bands were produced by POW on triplicates from all three samples. The lowest quality DNA was generated by CON with conspicuous degradation in both pig B and C.

All samples were successfully PCR-amplified with the exception of pig C extracted by CON, possibly due to protein or phenol contamination (A_260_/A_280_: 1.4), which can inhibit PCR [32]. CON has been previously described as an effective DNA isolation method from healthy multiparous Rongchang sow faeces [10] compared to QIAamp DNA Stool Mini Kit (QIAGEN, Duesseldorf, Germany) and three other conventional methods [33–35]. However, results show variation both in technical replicates, and across extraction methods. DNA yield, quality and success of PCR amplification have been previously used as DNA extraction method criteria [36–37]. Based on the combination of these three criteria, it would appear that FAS is the most consistent method to use for extracting DNA from porcine faeces (Fig 1).

### Pyrosequencing result comparison

**Fig 2:**
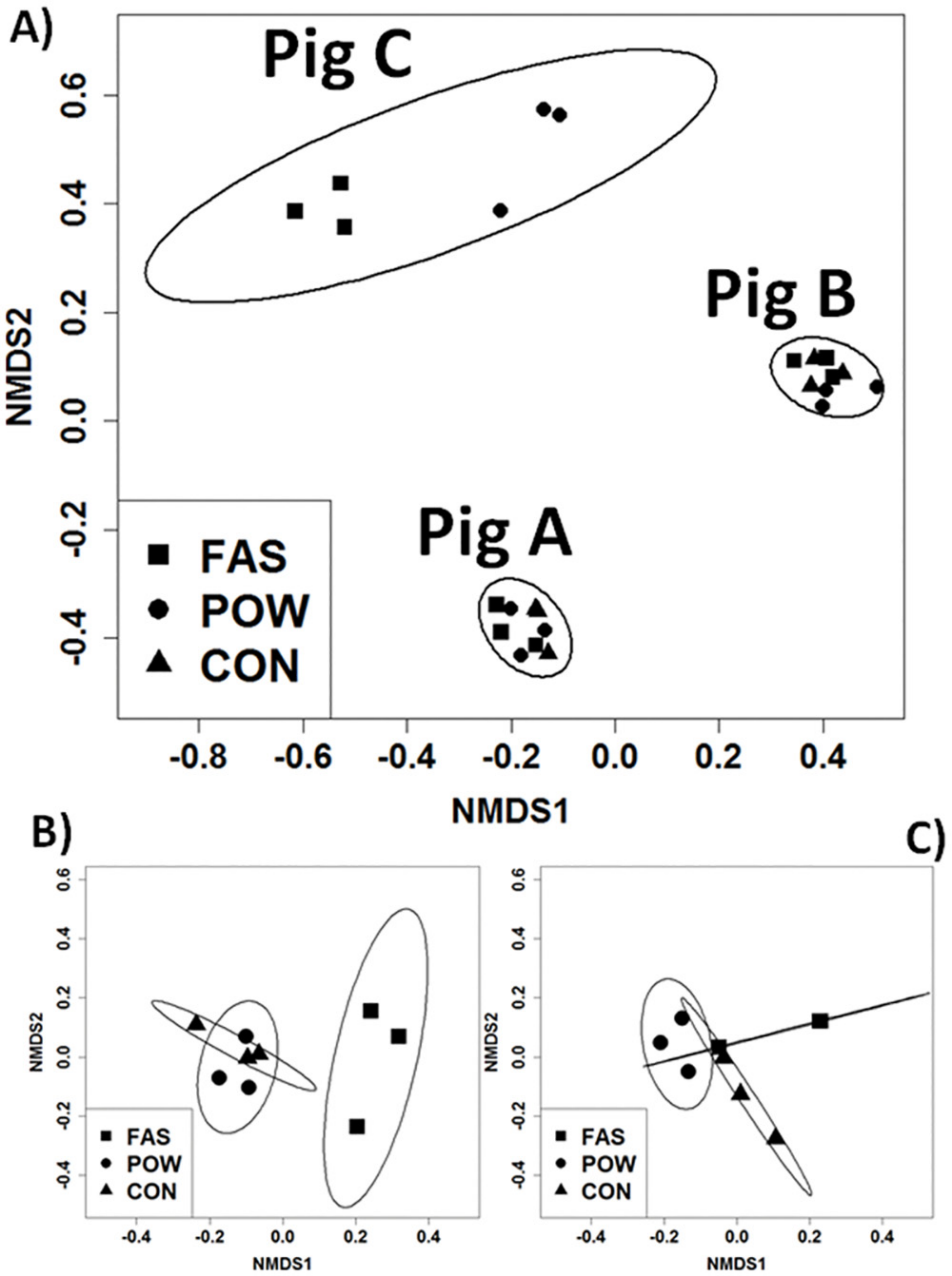
Nonmetric multidimensional scaling analysis of pyrosequencing result extracted with different methods between different pigs (A) within pig A (B) and within pig B (C). The replicates of each pig clustered well. Pyrosequencing results obtained from FAS differed to other methods in all pigs. Ellipses represent 95% prediction intervals (*s*_*x*_ *t*_*0*.*025*,*n-1*_), including correlation for host (A) or methods (B & C).

Pyrosequencing generated 132,984 high quality reads after quality filtering, which were grouped into 1,065 operational taxonomic units (OTUs). FAS resulted in lower numbers of OTUs from the same sample, especially from pig C (191±11, standard error), numbers of OTUs from other samples are listed in S2 Table. Nonmetric Multi-Dimensional Scaling (NMDS) analysis of the sequence data (weighted UniFrac distances) showed primary separation of microbial communities according to ANOVA by host (**R** ^**2**^ = 0.94, ***p*** = 5.2×10^−12^, with a power of 0.98) and secondarily by DNA extraction method (***p*** = 7.6×10^−03^) (Fig 2A). Separation within factors as analysed by ANOSIM was also strong with separation factors for host (R = 0.7, ***p*** = 0.001), and DNA extraction method (R = 0.2, ***p*** = 0.005).

**Fig 3:**
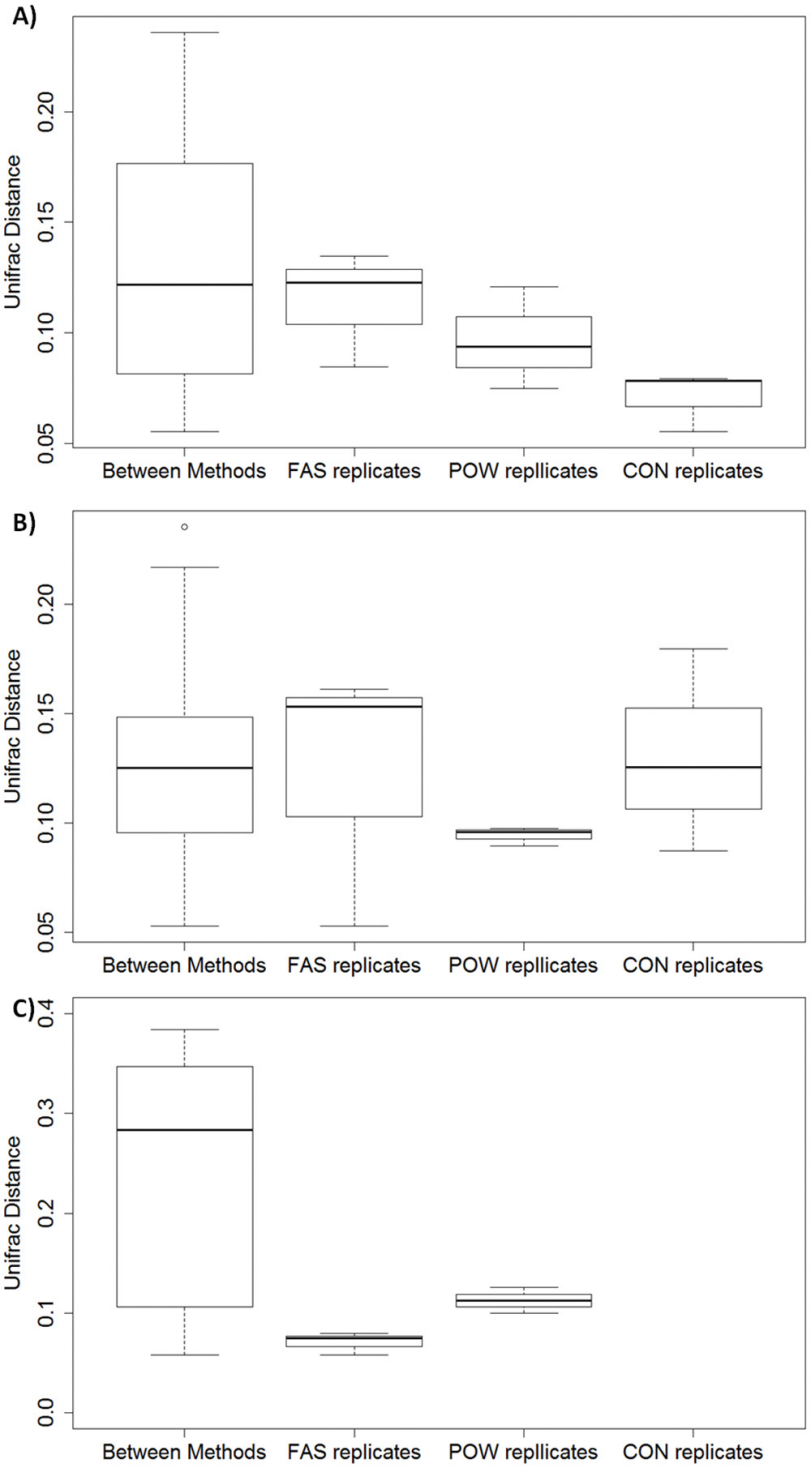
Boxplot of weighted UniFrac distance between methods and replicate extraction by the same method from pig A (A) B (B) and C (C). CON produces stable and reasonable (lowest in both pig A and B) variation in community profile between replicates.

Differences between replicates from the same extraction method were generally smaller than the differences between different extraction methods (ANOSIM: R = 0.5, ***p*** = 0.03 for pig A; R = 0.5, ***p*** = 0.02 for pig B) (Fig 2B and 2C). The same conclusion was found by evaluating different extraction methods on mock and bronchoalveolar lavage samples [38]. FAS had the highest variability in technical replicates in pig A and B fed with mainly wheat fines (Fig 3A and 3B). Although the UniFrac distances between replicates extracted by FAS from pig C was the lowest compared to POW and differences between methods (Fig 3C), it produced smeared gels (S1 Fig) and recovered the least OTUs indicating partial destruction of the DNA. Reproducibility from CON was also not stable as indicated by a large interquartile range (IQR, Fig 3B). CON utilized CTAB as the major lysis reagent in addition to bead beating and lysozyme (which were also used in both POW and FAS), which has been previously shown to generate variability in technical replicates [11]. In general, POW resulted in a lower range within replicates, and with a lower IQR, but this was not consistent for all hosts. In addition, pig C had the highest UniFrac distance and IQR indicating large variation between DNA extracted from FAS and POW. The major components in diet fed to pig C are barley fines which are physically more granular and abrasive than wheat fines [39], and thus possibly contribute to additional physical shear in addition to the extraction method.

**Fig 4:**
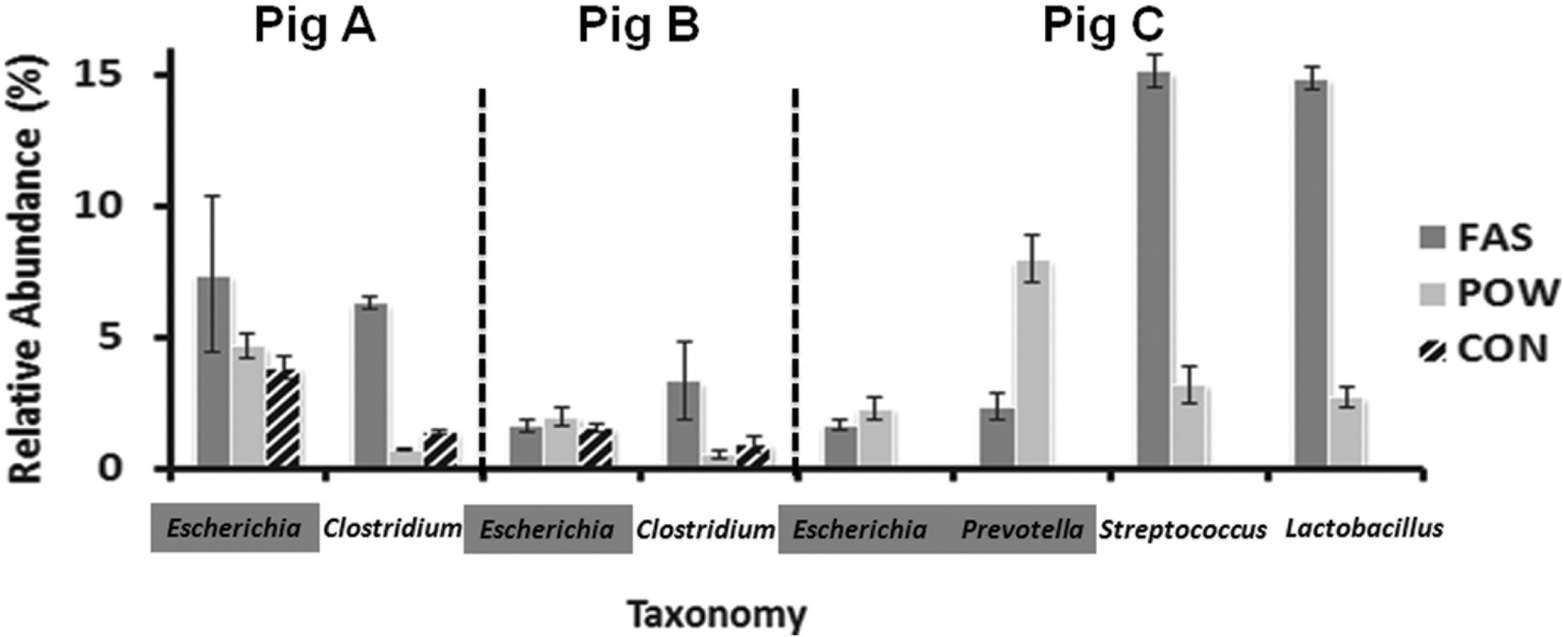
Mean abundance of specific Gram-positive (non-shaded) and Gram-negative (shaded) OTUs with large differences between difference methods. DNA extracted by FAS show huge variation on individual OTUs and between other methods.

Specific OTUs in the profiled communities can be identified which represent easier (Gram-negative) and harder (Gram-positive) to lyse populations (Fig 4). Consistent with NMDS analysis of microbial profiles from individual hosts (Fig 2B and 2C), POW and CON produced similar OTU relative abundances with FAS being the outlier method, increasing relative abundance of Gram-positive organisms and decreasing that of Gram-negatives (Fig 4), Abundances of Gram-positive bacteria recovered from FAS were often higher than other methods, which is likely due to two factors: 1. Because of thicker cell walls, and large amounts of peptidoglycan, Gram-positive bacteria are difficult to physically lyse. FAS is a harsher method and hence able to recover more Gram-positive microbes. 2. The abundance of Gram-negative bacteria such as *Prevotella* (of pig C in Fig 4) is relatively lower due to DNA destruction and hence leads to a relative increase in Gram-positive bacteria such as *Clostridium*, *Streptococcus*, and *Lactobacillus*. Similar effects were identified in other pigs with low abundance (S3 Table). The latter is likely, as FAS recovered the least OTUs and produced visible smeared gels from pig C. The same reduction on *Escherichia* (Gram-negative bacterium) can be observed to a lesser extent, except from pig A due to an outlier replicate with extreme high abundance (13% compared to 3 and 4% in other replicates of pig A, S3 Table), indicating a high variability between replication by FAS. Therefore, although FAS appeared to be the best method in terms of yield purity and PCR amplifiability (Fig 1), community profiling data suggest that POW produces less bias and should be the preferred method. A harsher single extraction such as FAS may be preferable for ensuring hard to lyse populations are accessed, but less suitable for objective community assessment. While CON was the only non-commercial method, it was the least suitable in general for these samples, and the cost of commercial kits is relatively minor in comparison with sampling and sequencing costs.

## Conclusions

The main factor determining microbial community profile variation is host, rather than DNA extraction method, indicating that the tested extraction methods likely do not substantially skew community composition. Similar conclusions were reported in human gut microbiota profiling [40]. Samples from which extraction is difficult (because of the matrix, i.e. pig C fed on barley) can be extracted using a harsher method, but variability in replicates, and between extraction methods increases, and can possibly lead to overestimation of the relative abundance of Gram-positive bacteria. The PowerSoil^®^ DNA Isolation Kit (POW), which extracts moderate yields of high quality DNA without apparently over-representing Gram-positive OTUs, is recommended for community profiling of pig faecal samples.

## Supporting Information

S1 Fig Example of DNA quality from different methods (FAS, POW and CON) and pigs (A, B and C) as shown in 2D-gel electrophoresis. (TIF)

S1 Table Proportions of feed ingredients for pigs. (DOCX)

S2 Table Number of OTUs recovered from each sample. (DOCX)

S3 Table Relative abundance of OTUs in the replicates of pigs. (XLSX)
