# Supplementary figures and images for: Evaluating DNA Extraction Methods for Community Profiling of Pig Hindgut Microbial Community
Source: PLoS One. 2015 Nov 11;10(11):e0142720. doi: 10.1371/journal.pone.0142720 (PMC4641665; doi:10.1371/journal.pone.0142720)

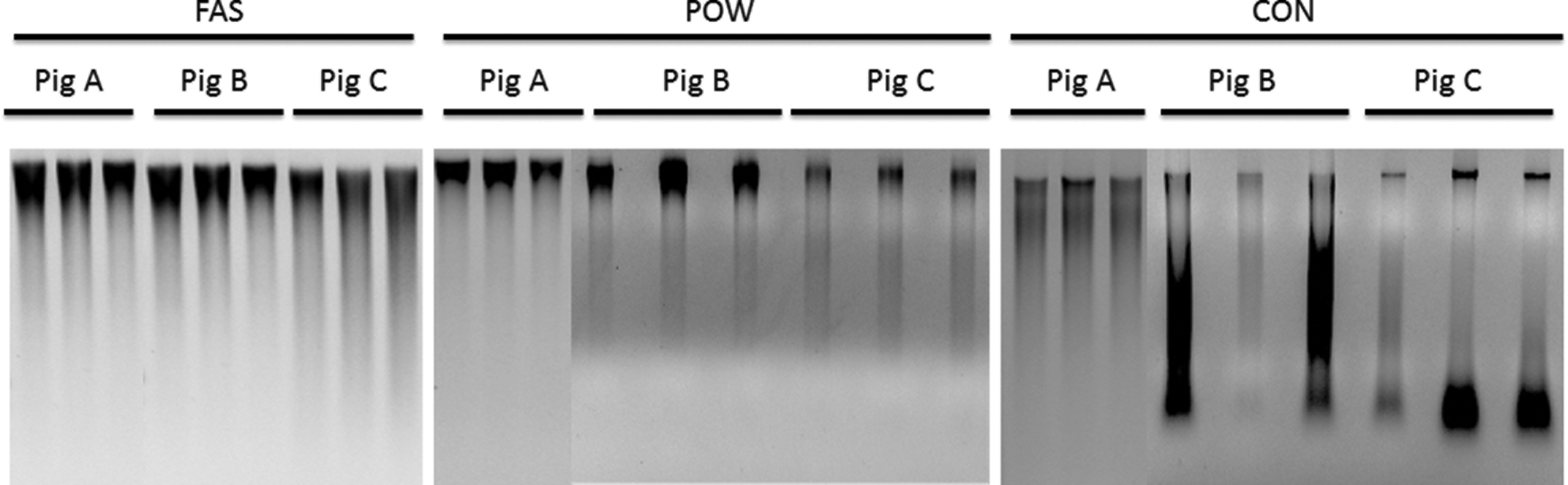

Supplement: S1 Fig — (TIF) [file pone.0142720.s001.tif]
