# Supplementary material for: Evaluating DNA Extraction Methods for Community Profiling of Pig Hindgut Microbial Community
Source: PLoS One. 2015 Nov 11;10(11):e0142720. doi: 10.1371/journal.pone.0142720 (PMC4641665; doi:10.1371/journal.pone.0142720)
Supplement: S1 Table — (DOCX) [file pone.0142720.s002.docx]

| **Raw material** | **Proportion of ingredient fed (% mass as-fed)** | | |
| --- | --- | --- | --- |
|  | **Pig A** | **Pig B** | **Pig C** |
| **Wheat Fine 13.5** | 72.23 | 20.13 | 20 |
| **Barley Fine 2 (Phantom) 11.5** | 10 |  | 48.09 |
| **Sorghum Fine 10.0** |  | 54.73 |  |
| **Meat Meal 51.0** | 5 | 5 | 4 |
| **Mung Beans 22.0** |  |  | 20 |
| **Tallow In Mixer** | 0.6 | 0.53 | 3.6 |
| **Canola Meal 37.0** | 4 | 11.93 |  |
| **Soybean Meal (Imp) 47.0** | 6.67 | 6.2 |  |
| **Full Fat Soyabean 38.0** |  |  | 2.67 |
| **Limestone Fine** | 0.67 | 0.67 | 0.67 |
| **Salt** | 0.2 | 0.2 | 0.2 |
| **Choline Chloride 70%** | 0.01 | 0.02 |  |
| **DL-Methionine** | 0.02 | 0.02 | 0.13 |
| **L-Lysine HCL** | 0.32 | 0.32 | 0.3 |
| **L-Threonine** | 0.04 |  | 0.11 |
| **Rap Pig Grw/Fin Pmx 2kgpt** | 0.2 | 0.2 | 0.2 |
| **Phyzyme XP5000 Pigs** | 0.01 | 0.01 | 0.01 |
| **Ronozyme WX CT** | 0.03 | 0.03 | 0.03 |
| **Total:** | 100 | 100 | 100 |
