# Supplementary material for: Evaluating DNA Extraction Methods for Community Profiling of Pig Hindgut Microbial Community
Source: PLoS One. 2015 Nov 11;10(11):e0142720. doi: 10.1371/journal.pone.0142720 (PMC4641665; doi:10.1371/journal.pone.0142720)
Supplement: S2 Table — (DOCX) [file pone.0142720.s003.docx]

| **Extraction Method** | **Number of OTUs ± standard error** | | |
| --- | --- | --- | --- |
|  | **Pig A** | **Pig B** | **Pig C** |
| **FAS** | 247±5 | 264±3 | 191±11 |
| **POW** | 270±9 | 269±18 | 240±15 |
| **CON** | 280±4 | 268±16 |  |
